# Seropositivity and history of hospitalisation for dengue in relation to anthropometric indices among Colombian children and adults

**DOI:** 10.1017/S0950268821000388

**Published:** 2021-02-15

**Authors:** M. R. Barry, L. A. Villar, O. F. Herrán, A. Lozano-Parra, M. I. Estupiñán, V. M. Herrera, E. Villamor

**Affiliations:** 1 Department of Epidemiology, University of Michigan School of Public Health, Ann Arbor, MI, USA; 2 Centro de Investigaciones Epidemiológicas, Facultad de Salud, Universidad Industrial de Santander, Bucaramanga, Colombia

**Keywords:** Anthropometry, body mass index, dengue, height, waist circumference

## Abstract

The role of anthropometric status on dengue is uncertain. We investigated the relations between anthropometric characteristics (height, body mass index and waist circumference (WC)) and two dengue outcomes, seropositivity and hospitalisation, in a cross-sectional study of 2038 children (aged 2–15 years) and 408 adults (aged 18–72 years) from Bucaramanga, Colombia. Anthropometric variables were standardised by age and sex in children. Seropositivity was determined through immunoglobulin G antibodies; past hospitalisation for dengue was self-reported. We modelled the prevalence of each outcome by levels of anthropometric exposures using generalised estimating equations with restricted cubic splines. In children, dengue seropositivity was 60.8%; 9.9% of seropositive children reported prior hospitalisation for dengue. WC was positively associated with seropositivity in girls (90th *vs.* 10th percentile adjusted prevalence ratio (APR) = 1.19; 95% confidence interval (CI) 1.03–1.36). Among adults, dengue seropositivity was 95.1%; 8.1% of seropositive adults reported past hospitalisation. Height was inversely associated with seropositivity (APR = 0.90; 95% CI 0.83–0.99) and with hospitalisation history (APR = 0.19; 95% CI 0.04–0.79). WC was inversely associated with seropositivity (APR = 0.89; 95% CI 0.81–0.98). We conclude that anthropometry correlates with a history of dengue, but could not determine causation. Prospective studies are warranted to enhance causal inference on these questions.

## Introduction

Dengue virus (DENV) is the most common mosquito-borne virus in the world, where up to 3.9 billion people are at risk of infection [1]. The burden of disease primarily resides in tropical and subtropical regions across Asia, Africa and Latin America, where an estimated 390 million infections, 96 million clinical events [2] and 13 000 deaths [3] occur each year. Dengue is highly endemic in several regions of Colombia [4]. Because no preventive or curative treatment exists for dengue infection or disease, the identification of modifiable risk factors is a high research priority.

The nutritional status could modulate the risk and response to infections, and some evidence indicates that it may be relevant in the case of dengue. Both micronutrient status [5–9] and long-term protein and energy balance as measured with anthropometric indices [10, 11] have been associated with dengue-related outcomes in epidemiologic studies. Nevertheless, the results have not always been consistent, particularly concerning anthropometric exposures. Most studies have focused on the role of anthropometry on progression to severe dengue disease in children. Based on observations in dengue endemic regions, experts once thought that protein-energy malnutrition might protect against severe disease through dampening immune and inflammatory responses [12]. Evidence from some paediatric cross-sectional and case-control studies supported this view. For example, underweight (low weight-for-age) and stunting (low height-for-age) were inversely associated with severe disease in children from Vietnam [13] and Thailand [14], whereas anthropometric indices of overweight and obesity were positively related to disease severity in children from Thailand [12, 15] and Indonesia [16]. However, findings from other studies refuted the hypothesis. Underweight was positively associated with severe disease in a large case-control investigation of Thai children [17] and unrelated in other studies from India [18], El Salvador [19], Indonesia [20], Thailand [21], Cuba [22] or Sri Lanka [23]. In addition, overweight was unrelated to disease severity in various settings [17, 19, 21].

Few studies have aimed to identify nutritional risk factors for dengue infection. Anthropometric characteristics might conceivably modify infection risk through mechanisms influencing the efficiency of mosquito bites or as proxies for the immunological fitness required to mount a humoral response against the initial viral infection. The role of anthropometric status on dengue-related outcomes has not been systematically examined in adults. We conducted a cross-sectional investigation in a large sample of children and adults from Colombia to study the associations of anthropometric indices with two dengue-related outcomes; history of infection according to anti-DENV immunoglobulin G (IgG) titres, and history of severe dengue disease per the self-report of previous hospitalisation for dengue.

## Methods

### Study population and field methods

We conducted a cross-sectional investigation using data obtained at the time of recruitment into a cohort study that aimed to identify optimal dengue vaccination strategies. The study was conducted in Piedecuesta, a municipality within the metropolitan area of the city of Bucaramanga in northeast Colombia, located at an average altitude of 1005 m (range 600–3600) a.s.l., with mean temperature 24 °C, relative humidity 72%, and an estimated population of 152 448 by 2015. This town is epidemiologically similar to other dengue endemic regions in Colombia. We implemented different convenience sampling strategies to maximise enrolment of children into the study. First, we identified seven neighbourhoods where the town's schools and daycare centres were located and five additional neighbourhoods with the highest participation rates in a prior investigation on dengue. We aimed at recruiting 2000 children aged 2–15 years and 400 adults (≥18 year-old) from these neighbourhoods, in proportion to each neighbourhood's population size for these age groups.

Prior to recruitment, we provided educational materials about dengue infection and the upcoming study in the target neighbourhoods. Between June and October 2015, trained research assistants visited the neighbourhoods' households door-to-door, inviting children's caregivers to participate. In each household with at least one child, we offered enrolment to all eligible children plus one adult until the target sample size proportional to each neighbourhood's population was reached. Among those who agreed to participate (85%), we sought written informed consent; parents or primary caregivers provided consent for their children and adults consented for themselves. Assent to participate was confirmed verbally from children aged ≥7 years. The Universidad Industrial de Santander Ethics Committee approved the study protocol and procedures. The University of Michigan Institutional Review Board approved the use of data from the study.

At the recruitment visit, we conducted an interview with an adult household informant to elicit information on sociodemographic characteristics and known history of dengue infection. Sociodemographic data included the participant's birth date, the informant's education level, family monthly income in minimum wage multiples, home ownership, housing characteristics and number of residents and the socioeconomic status (SES) classification according to the local government's rating for public services fees. Questions pertaining to dengue history involved whether the participant had ever been infected with dengue, whether this infection had been diagnosed by a physician, and whether they had ever been hospitalised for dengue. Anthropometry was performed according to standardised procedures using calibrated instruments. Height was measured without shoes to the nearest centimetre using a Seca 213 portable stadiometer (Seca, Hanover, MD), weight was measured in light clothing and without shoes to the nearest 100 g using a Kenwell EF-432-BW digital scale (Badecol, Cali, Colombia), and waist circumference (WC) was measured at the midpoint between the lowest rib and the iliac crest to the nearest centimetre using a non-extendable Seca 201 measuring tape (Seca, Hanover, MD). At the end of the visit, research assistants obtained blood samples by antecubital venepuncture; 5 ml were collected in a tube without anticoagulant for separation of serum. The samples were placed in insulated containers (Giostyle Spa, Urgnano, Italy) precooled at 2–8 °C, that were monitored at every opening. They were transported on the same day of collection to the study laboratory (Parque Tecnológico Guatiguará) where serum was separated by centrifuging at 3500 rpm/10 min and aliquoted.

### Laboratory methods

We quantified serum anti-DENV IgG with use of the indirect enzyme-linked immunosorbent assay (ELISA) Panbio kit (Alere, Australia). The test's sensitivity is 96% and the specificity ranges from 91% to 100%.

### Data analysis

We included in the analyses all 2038 children and 408 adults recruited. Among children, the informant was the mother, father or another guardian in 89.4%, 10.2% and 0.5%, respectively.

There were two primary outcomes: IgG seropositivity defined as a Panbio test result >11 units, and, among seropositive participants only, past hospitalisation for dengue according to their self-report.

Primary exposures for children included height-for-age *Z* score (HAZ), BMI-for-age *Z* score (BAZ) and waist circumference-for-age *Z* score (WCZ). HAZ and BAZ were estimated through standardisation by age and sex according to the World Health Organization (WHO) growth standards for children aged <5 years and WHO growth references for children aged 5–19 years. For WCZ, we used the LMS method [24] to standardise WC by age according to sex-specific WC data for children aged 2–16 years in the third National Health and Nutrition Examination Survey (NHANES III). Briefly, the LMS method uses a smoothed function of the Box-Cox power (L) to normalise measurement distributions across age groups in a population. Trends in mean (*M*) and coefficient of variation (*S*) across age-group distributions are also smoothed, allowing the calculation of age-specific *Z* scores. To obtain a measure of abdominal adiposity independent of overall adiposity, we further adjusted WCZ for BAZ using the method of residuals [25, 26]. We fitted a linear regression model with WCZ as the outcome and BAZ as the exposure; the residuals output from the model were used as a measure of variability in WCZ after removing inherent variation due to BAZ. We added a constant (the mean WCZ predicted by the linear regression model at the mean BAZ in the population) to the raw residual values to improve interpretability. For adults, exposures included height, BMI and WC; WC was adjusted for BMI using the method of residuals.

Covariates considered included sex, age and SES indicators such as the informant's education level, family income, home ownership, housing type, number of people per room at home and the household's socioeconomic classification per the local government's rating. Age was calculated by subtracting date of birth from date of assessment. Number of people per room was estimated by dividing the household's number of residents by the number of rooms.

Statistical analyses were performed separately for children and adults. In bivariate analysis, we compared the prevalence of seropositivity and the prevalence of past hospitalisation for dengue by categories of sociodemographic characteristics. We estimated prevalence ratios from generalised estimating equations (GEE) with the Poisson distribution, using the robust sandwich estimate of the variance to account for intra-family correlations. For ordinal predictors, we conducted tests for linear trend by introducing into the GEE models a variable representing median values of the ordinal categories as a continuous predictor.

Next, we compared the prevalence of seropositivity and prevalence of past hospitalisation by anthropometric indices with use of GEE models with restricted cubic splines. Cubic splines represent non-linear terms for the anthropometric exposures that allow smoothing of potential non-linear relations between exposure and outcome. Piece cubic polynomials are smoothly connected at joint points or ‘knots’ [27]. Knots were placed at the 5th, 25th, 50th, 75th and 95th percentiles of each anthropometric variable. In each model, the outcome was seropositivity or past hospitalisation for dengue and covariates comprised the linear and spline terms for the anthropometric exposure, plus adjustment variables. Selection of confounders was driven primarily by prior causal knowledge of independent predictors of arboviral infection and correlates of anthropometric exposures that were not their consequence. These included sex, age and SES indicators [28]. We favoured those indicators that significantly predicted outcomes in this population at *P* < 0.05. Because there were very few missing values on covariates, adjusted analyses were complete case. The spline functions were centred at *Z* score values of 0 in children and at the medians of each anthropometric characteristic distribution in adults. We computed adjusted prevalence ratios (APRs) with 95% confidence intervals (CI) for each outcome by comparing the prevalences estimated from the non-linear cubic spline models at the 90th *vs.* the 10th percentiles of each anthropometric variable distribution. In supplemental analyses, we estimated prevalence ratios per unit of each anthropometric indicator, assuming linearity. Among children, we tested for interactions between anthropometric exposures and sex with use of the likelihood ratio test. All analyses were conducted using Statistical Analysis Software version 9.4 (SAS Institute, Cary, NC).

## Results

### Children

Mean ± s.d. age was 8.8 ± 3.7 years; 49.7% were girls. Mean ± s.d. HAZ, BAZ and WCZ were −0.30 ± 1.02, 0.25 ± 1.27 and −0.13 ± 0.68, respectively; 4.9% were stunted (HAZ < –2), 3.6% were underweight (BAZ < –2) and 26.6% were overweight or obese (BAZ > 1). Dengue IgG seroprevalence was 60.8%. Seropositivity was positively related to age and home ownership and inversely associated with the informant's education level (Table 1). Among 1111 seropositive children with non-missing past hospitalisation status, 9.9% had been hospitalised for dengue. Hospitalisation was positively associated with the informant's education level and home ownership, and inversely related to the number of people per room at home (Table 1).

**Table 1.** Prevalence of IgG seropositivity and past hospitalisation for dengue by sociodemographic characteristics in children from Bucaramanga, Colombia

Characteristic	Seropositive			Hospitalised^a^		
	*n*	%	Prevalence ratio (95% CI)^b^	*n*	%	Prevalence ratio (95% CI)
Sex						
Female	1012	61.0	1.00	555	9.6	1.00
Male	1026	60.7	1.00 (0.93–1.07)	556	10.3	1.07 (0.75–1.55)
*P*^c^			0.91			0.70
Age group, years						
2 to <5	387	32.0	1.00	111	10.8	1.00
5 to <11	1003	59.0	1.84 (1.58–2.15)	522	10.2	0.94 (0.52–1.70)
11 to <16	648	80.9	2.52 (2.17–2.94)	478	9.4	0.87 (0.47–1.60)
*P*, trend^d^			<0.0001			0.61
Informant's education^e^						
Primary or less	669	67.6	1.00	410	6.1	1.00
Secondary	1278	58.0	0.86 (0.79–0.93)	655	11.9	1.95 (1.25–3.06)
University	82	51.2	0.76 (0.60–0.95)	41	17.1	2.80 (1.12–6.99)
*P*, trend			<0.0001			0.001
Missing	9	55.6		5	0.0	
Monthly family income						
<1 min wage salary	664	61.6	1.00	370	9.5	1.00
1–2 min wage salaries	679	61.7	1.00 (0.91–1.10)	357	9.2	0.98 (0.61–1.57)
≥3 min wage salaries	126	60.3	0.98 (0.83–1.16)	70	11.4	1.21 (0.59–2.47)
*P*, trend			0.88			0.77
Missing	569	59.1		314	10.8	
Home ownership						
No	1572	59.5	1.00	832	8.4	1.00
Yes	458	65.3	1.10 (1.01–1.20)	274	14.6	1.74 (1.19–2.54)
*P*			0.03			0.004
Missing	8	75.0		5	0.0	
Housing type						
House or apartment	1314	60.4	1.00	713	10.4	1.00
One room or multifamily	719	61.8	1.02 (0.94–1.11)	395	9.1	0.88 (0.59–1.30)
*P*			0.57			0.52
Missing	5	60.0		3	0.0	
People per room						
1	184	65.8	1.00	112	14.3	1.00
2	1252	61.2	0.93 (0.82–1.05)	681	11.2	0.78 (0.48–1.27)
3	454	59.0	0.90 (0.78–1.03)	238	5.5	0.38 (0.19–0.76)
>3	143	57.3	0.87 (0.72–1.05)	77	6.5	0.45 (0.18–1.15)
*P*, trend			0.16			0.02
Missing	5	60.0		3	0.0	
SES^f^						
1 or 2 (lower)	1199	62.7	1.00	660	9.1	1.00
3 or 4	834	58.2	0.93 (0.86–1.01)	448	11.2	1.23 (0.85–1.78)
*P*			0.07			0.28
Missing	5	60.0		3	0.0	

a Among IgG seropositive children only.

b From GEE with the Poisson distribution. Seropositivity or hospitalisation was the dichotomous outcome and indicator variables for each characteristic were predictors. In all models, the robust sandwich estimate of the variance was used to account for intra-family correlations.

c Wald test.

d Wald test for a variable representing ordinal categories of the predictor entered into the model as a continuous covariate.

e Informant: mother, 89.4%; father, 10.2%; other guardian, 0.5%.

f According to the local government classification for public services fees.

**Fig. 1.**
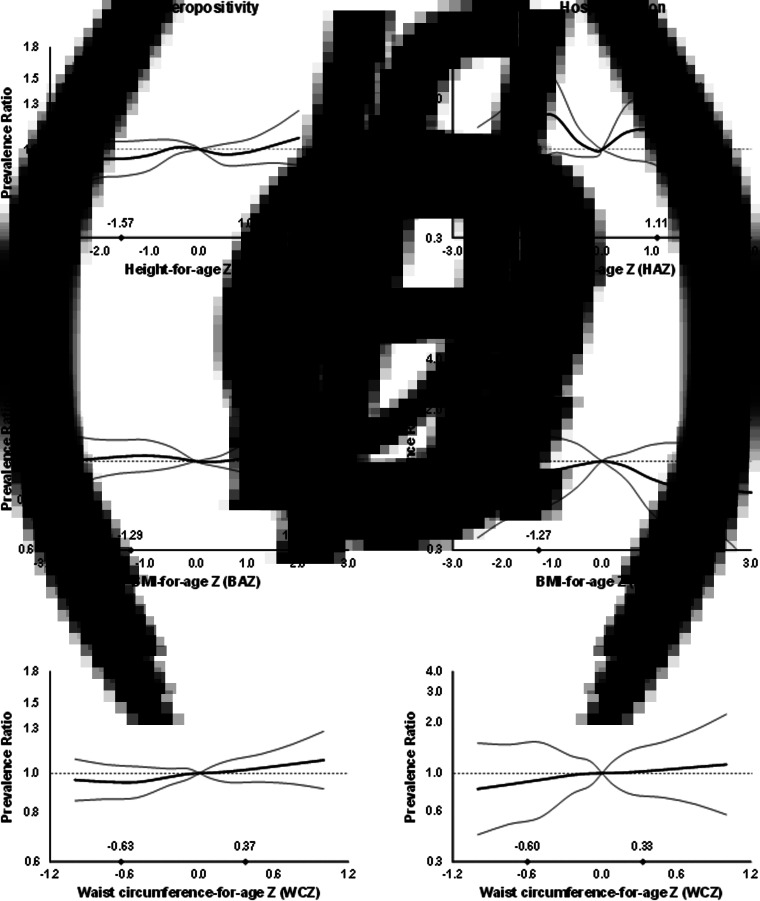
Prevalence ratios for IgG seropositivity (a, b and c) and past hospitalisation for dengue (d, e and f) by anthropometric characteristics in children from Bucaramanga, Colombia. Diamonds indicate 10th and 90th percentiles for anthropometric indices. WCZ was adjusted for BAZ using the method of residuals. Estimates are from GEE with the Poisson distribution; seropositivity or past hospitalisation was the dichotomous outcome and predictors included linear and spline terms for each anthropometric index plus indicator variables for age, sex, informant's education level and home ownership. Models for BAZ and WCZ included both BAZ and BAZ-adjusted WCZ as covariates. In all models, the robust sandwich estimate of the variance was used to account for intra-family correlations. Estimates for hospitalisation are restricted to IgG seropositive children.

**Fig. 2.**
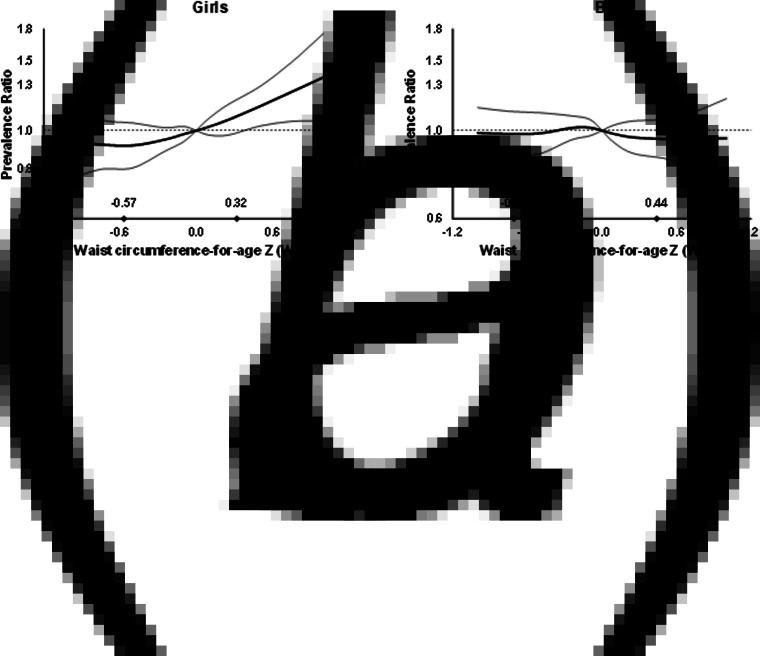
Prevalence ratios for IgG seropositivity by WCZ in girls (a) and boys (b) aged 2–15 years from Bucaramanga, Colombia. WCZ was adjusted for BAZ using the method of residuals. Diamonds indicate 10th and 90th percentiles of BAZ-adjusted WCZ. Estimates are from GEE with the Poisson distribution; seropositivity was the dichotomous outcome and predictors included linear and spline terms for BAZ-adjusted WCZ, BAZ, plus indicator variables for age, informant's education level and home ownership. The robust sandwich estimate of the variance was used to account for intra-family correlations. *P*, test for interaction between WCZ and sex = 0.02.

In multivariable analysis, there was no association between anthropometric indices and dengue outcomes in children overall (Fig. 1, Supplementary Table S1). However, WCZ was positively associated with seropositivity in girls (*P*, test for interaction = 0.02, Fig. 2, Supplementary Table S1). Girls at the 90th percentile of WCZ had a 19% higher adjusted prevalence than those at the 10th percentile (APR = 1.19; 95% CI 1.03–1.36; *P* = 0.01). Every unit WCZ was related to a 21% higher prevalence of seropositivity (APR = 1.21; 95% CI 1.04–1.39, *P* = 0.01) (Supplementary Table S2). There was no association in boys.

### Adults

Mean ± s.d. age of participants was 35.4 ± 9.7 years; 50.7% were women. Mean ± s.d. height, BMI and WC were 162.4 ± 8.8 cm, 26.6 ± 4.5 kg/m^2^ and 86.8 ± 11.4 cm, respectively; 2.0% were underweight (BMI < 18.5) and 63.5% were overweight or obese (BMI ≥ 25). The majority of adults (95.1%) were dengue IgG seropositive. Seropositivity was not associated with the sociodemographic characteristics studied (Table 2). Among 344 seropositive adults with non-missing past hospitalisation status, 8.1% reported past hospitalisation for dengue; hospitalisation was inversely associated with SES (Table 2).

**Table 2.** Prevalence of IgG seropositivity and past hospitalisation for dengue by sociodemographic characteristics in adults from Bucaramanga, Colombia

Characteristic	Seropositive			Hospitalised^a^		
	*n*	%	Prevalence ratio (95% CI)^b^	*n*^c^	%	Prevalence ratio (95% CI)
Sex						
Female	207	95.7	1.00	177	6.8	1.00
Male	201	94.5	0.99 (0.95–1.03)	167	9.6	1.41 (0.71–2.83)
*P*^c^			0.58			0.33
Age group, years						
18 to <30	117	95.7	1.00	98	8.2	1.00
30 to <40	175	94.9	0.99 (0.94–1.05)	153	7.2	0.88 (0.36–2.17)
≥40	116	94.8	0.99 (0.94–1.05)	93	9.7	1.19 (0.47–2.96)
*P*, trend^d^			0.76			0.70
Education						
Primary or less	117	94.0	1.00	93	9.7	1.00
Secondary	278	95.3	1.01 (0.96–1.07)	240	7.5	0.78 (0.36–1.66)
University	13	100.0	1.06 (1.02–1.11)	11	9.1	0.94 (0.13–6.92)
*P*, trend			0.37			0.61
Monthly family income						
<1 min wage salary	113	94.7	1.00	95	4.2	1.00
1–2 min wage salaries	140	95.7	1.01 (0.96–1.07)	120	12.5	2.97 (1.01–8.71)
≥3 min wage salaries	36	91.7	0.97 (0.87–1.08)	29	6.9	1.64 (0.34–7.95)
*P*, trend			0.72			0.10
Missing	119	95.8		100	7.0	
Home ownership						
No	289	94.5	1.00	242	8.7	1.00
Yes	118	96.6	1.02 (0.97–1.08)	101	6.9	0.80 (0.35–1.82)
*P*			0.38			0.59
Missing	1	100.0		1	0.0	
Housing type						
House or apartment	258	95.0	1.00	212	7.6	1.00
One room or multifamily	150	95.3	1.00 (0.96–1.05)	132	9.1	1.20 (0.58–2.49)
*P*			0.87			0.62
People per room						
1	39	94.9	1.00	29	13.8	1.00
2	267	95.1	1.00 (0.93–1.09)	225	6.2	0.45 (0.16–1.27)
3	77	97.4	1.03 (0.95–1.11)	69	11.6	0.84 (0.28–2.51)
>3	25	88.0	0.93 (0.79–1.10)	21	9.5	0.69 (0.15–3.11)
*P*, trend			0.51			0.64
SES^e^						
1 or 2 (lower)	225	95.1	1.00	190	11.6	1.00
3 or 4	183	95.1	1.00 (0.95–1.05)	154	3.9	0.34 (0.14–0.81)
*P*			0.99			0.01

a Among IgG seropositive adults only.

b From GEE with the Poisson distribution. Seropositivity or hospitalisation was the dichotomous outcome and indicator variables for each characteristic were predictors. In all models, the robust sandwich estimate of the variance was used to account for intra-family correlations.

c Wald test.

d Wald test for a variable representing ordinal categories of the predictor entered into the model as a continuous covariate.

e According to the local government classification for public service fees tax and planning purposes.

**Fig. 3.**
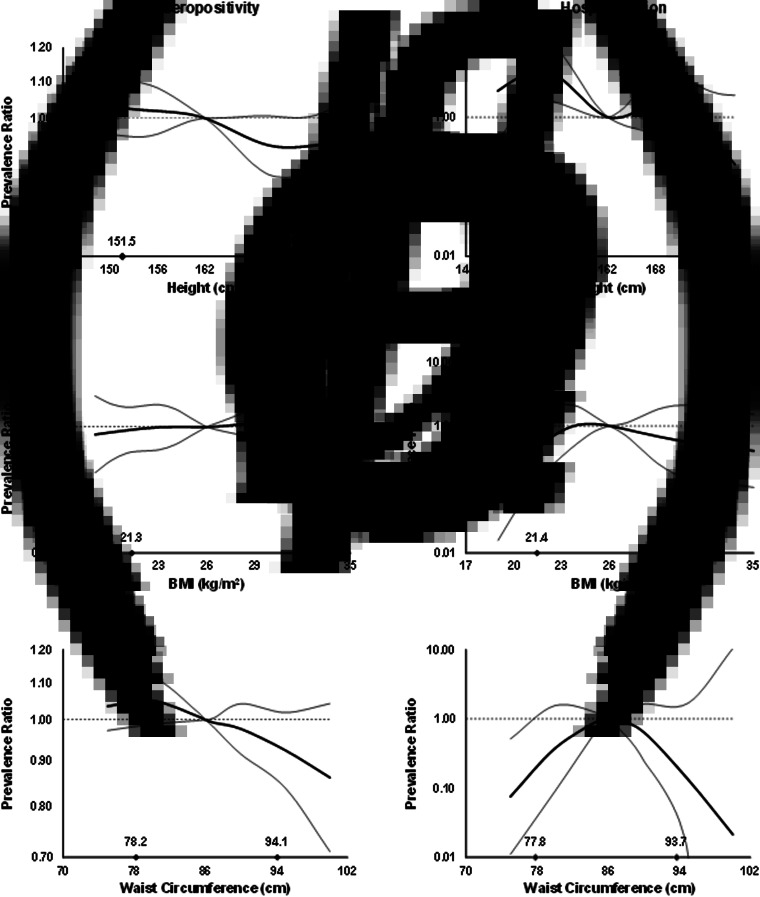
Prevalence ratios for IgG seropositivity (a, b and c) and past hospitalisation for dengue (d, e and f) by anthropometric characteristics in adults from Bucaramanga, Colombia. Diamonds indicate 10th and 90th percentiles for anthropometric indices. WC was adjusted for BMI using the method of residuals. Estimates are from GEE with the Poisson distribution; seropositivity or past hospitalisation was the dichotomous outcome and predictors included linear and spline terms for each anthropometric index plus indicator variables for age, sex, education level and SES. Models for BMI and WC included both BMI and BMI-adjusted WC as covariates. In all models, the robust sandwich estimate of the variance was used to account for intra-family correlations. Estimates for hospitalisation are restricted to IgG seropositive adults.

In multi-variable analysis, height was inversely associated with seropositivity (APR comparing the 90th *vs.* 10th height percentiles = 0.90; 95% CI 0.83–0.99; *P* = 0.03), as was WC (APR = 0.89; 95% CI 0.81–0.98; *P* = 0.02). Among seropositive adults, only height was inversely associated with hospitalisation (APR = 0.19; 95% CI 0.04–0.79; *P* = 0.02) (Fig. 3, Supplementary Table S3). Every centimetre of height was related to a 6% lower prevalence of hospitalisation history (APR = 0.94; 95% CI 0.90–0.99; *P* = 0.01) (Supplementary Table S4).

## Discussion

In this cross-sectional study, WCZ was positively associated with dengue IgG seropositivity in girls. In adults, height and WC were inversely associated with seropositivity. Among seropositive adults, height was inversely associated with past hospitalisation for dengue.

The positive association between WCZ, an indicator of central adiposity, and seropositivity for dengue in girls is a novel finding. Previous studies had compared indicators of overall adiposity between children hospitalised with severe dengue and uninfected children [16, 18]. One study in Thailand found that overweight, defined as high weight-for-age, was positively associated with hospitalisation for dengue infection [16]. However, weight-for-age is not a measure of central adiposity because it is influenced by height. Also, infected children without severe clinical manifestations were excluded from the study. It is uncertain why the association was apparent only in girls. One might speculate that a sex-specific effect could represent differential effects of abdominal obesity on synthesis of sex hormones, which may be related to antibody production. For example, high oestrogen and low testosterone concentrations have been positively related to Ig synthesis [29].

Our findings in adults are also novel. Little research exists on the associations between anthropometric measures and dengue outcomes in this age group. Height was inversely related to seropositivity despite the high prevalence of this outcome, and to past hospitalisation for dengue among adults who were seropositive. Poor linear growth has been related to compromised immunological fitness [30] and that might explain an inverse association between adult height, the end-result of child growth, and infection or severe disease. It is also possible that adult height represents other early-life exposures that result in enhanced immunity, such as energy balance or micronutrient intake. The findings may also have non-causal explanations. Because most infections leading to a positive IgG result are likely to happen before physical growth is completed, reverse causation could explain an inverse association of final height with seropositivity if early-life dengue infection stunts growth. Nevertheless, we did not find an association between height and seropositivity in children. Residual confounding by socioeconomic characteristics that are positively related to height and inversely to dengue infection could also explain the association. Longitudinal studies in children are warranted to disentangle the relation between linear growth and dengue-related outcomes.

In adults, WC was inversely related to dengue IgG seropositivity. This is in contrast to a study of Swedish adults, in whom WC was positively associated with seroprevalence for IgG antibodies against Sindbis virus, another arbovirus [31]. The inverse relation between adiposity and seropositivity in adults could be explained by impaired humoral immune function resulting from excess adiposity and chronic inflammation [32, 33]. Excess adiposity in the abdominal region may be more inflammatory than excess body fat generally [33]. Our finding of an inverse relation between adiposity and seropositivity is consistent with previous studies among adults indicating impaired antibody production in response to vaccination for hepatitis B [34] and influenza [35], possibly due to decreased antibody production in persons with higher fat mass [36, 37]. Since severe infection might result in acute weight loss, reverse causation may play a role, although we found no association between WC and past hospitalisation for dengue in adults. Of note, the association between WC and seropositivity was positive in girls but inverse in adults. It is difficult to reconcile these findings in the context of a cross-sectional study, especially since very few adults remained seronegative; it is not possible to discard that an effect of adiposity on risk of infection varies with age. We also noted that, in both children and adults, BMI, a measure of overall adiposity, was not associated with the outcomes. It may be that the effects of overall *vs.* abdominal adiposity on dengue-related outcomes differ.

Overall, associations of covariates with dengue seropositivity and hospitalisation history supported the internal validity of this study. In children, older age was associated with greater prevalence of dengue IgG seropositivity. This finding was expected since the number of infections in an endemic area should accumulate over time. Consistent with this notion, seropositivity was almost universal in adults. SES indicators were associated with one or both dengue outcomes among children. Of note, higher parental education was related to lower seropositivity but higher hospitalisation history prevalence. The seropositivity outcome was assessed in the whole population, whereas hospitalisation was only evaluated among seropositive children; thus, underlying differences between seronegative and seropositive children might explain differences in the direction of the estimates. Another potential explanation is that the inverse association of education with seropositivity reflects greater prevention knowledge among the more educated, whereas a positive association with hospitalisation might be related to increased awareness of danger severity signs, leading to medical care seeking behaviour. In adults, SES was inversely associated with hospitalisation history, which might reflect the influence of unmeasured factors among the better-off that reduce risk of progression to severe disease.

Our study has many strengths. The large sample size provided adequate statistical power to examine the questions of interest, especially in children. We used an objective measure to assess whether participants had ever been infected with DENV, which precludes recall bias. In addition, we had an opportunity to differentiate the associations of overall *vs.* abdominal adiposity with dengue outcomes by using different anthropometric indicators. The use of smoothing analytic techniques allowed us to explore non-linear associations between anthropometric measures and dengue outcomes.

Some limitations are also worth noting. First, from the cross-sectional design it was not possible to ascertain when dengue IgG seroconversion or hospitalisation occurred. Thus, reverse causation bias could explain the findings if dengue-related outcomes influenced anthropometric characteristics before the time of the study. Second, past hospitalisation for dengue was self-reported and could have resulted in misclassification due to recall bias. Because information on socioeconomic indicators was self-reported, there may also be a possibility of misclassification due to social desirability bias. The effect of this bias on the estimates of association would depend on whether it was differential or not with respect to exposure and outcome, and cannot be easily anticipated without further assumptions. Third, the very high prevalence of seropositivity in adults may have reduced statistical power. Fourth, analysing a large number of exposures may increase type I error, so we cannot rule out chance as a possible explanation for the observed associations. Finally, the ELISA IgG antibody test is an imperfect proxy for previous dengue infection because it can cross-react with antibodies resulting from infection with other flaviviruses, such as yellow fever and Zika viruses [38]. However, there has not been an outbreak of yellow fever in the Bucaramanga area since 1923 [39] and Zika virus did not exist in Colombia at the time of recruitment into the study [40, 41]. Thus, it is unlikely that our data are significantly affected by cross-reactivity due to previous yellow fever or Zika infections. No other flaviviruses circulate in this region. Cross reactivity with the yellow fever vaccine is also possible [38]. Vaccination against yellow fever was included in the expanded programme for immunisation of Colombia only in 2002, with one dose recommended at 12 months of age [42, 43]. Thus, study participants born in 2002 or later should be more likely to have received the yellow fever vaccine than those born before. Yet, seropositivity in participants born in 1999 or 2000 (108 of 119 participants, or 90.8%) was higher than that of participants born in 2002 or 2003 (222 of 286 participants, or 77.6%). These data suggest that seropositivity due to cross-reactions with the yellow fever vaccine in our study should be low.

In conclusion, WCZ is positively associated with dengue seropositivity in girls. Conversely, height and WC are inversely associated with seropositivity in adults, and height is inversely associated with past hospitalisation in seropositive adults. Prospective cohort studies are warranted to better understand the associations between anthropometric characteristics and dengue outcomes in both children and adults.

## Data Availability

The data that support the findings can be made available by the corresponding author upon a reasonable request.

## Supplementary material

For supplementary material accompanying this paper visit http://dx.doi.org/10.1017/S0950268821000388. click here to view supplementary material
