# Supplementary material for: Seropositivity and history of hospitalisation for dengue in relation to anthropometric indices among Colombian children and adults
Source: Epidemiol Infect. 2021 Feb 15;149:e58. doi: 10.1017/S0950268821000388 (PMC8060849; doi:10.1017/S0950268821000388)
Supplement: Supplementary file 1 [file S0950268821000388sup001.docx]

Supplementary Table S1. Unadjusted and adjusted prevalence ratios for IgG seropositivity and past hospitalization for dengue by anthropometric characteristics in children from Bucaramanga, Colombia, assuming non-linearity.

| Anthropometric characteristic | Seropositive  Prevalence Ratio (95% CI)^1^ | |  | Hospitalized  Prevalence Ratio (95% CI)^1,2^ | |
| --- | --- | --- | --- | --- | --- |
|  | Unadjusted | Adjusted^3^ |  | Unadjusted | Adjusted^3^ |
|  |  |  |  |  |  |
| Height-for-age Z |  |  |  |  |  |
| Overall | 1.05 (0.95, 1.16) | 1.04 (0.95, 1.14) |  | 1.05 (0.63, 1.75) | 0.94 (0.57, 1.55) |
| Girls | 1.07 (0.93, 1.22) | 1.09 (0.96, 1.24) |  | 0.93 (0.46, 1.86) | 0.83 (0.42, 1.65) |
| Boys | 1.02 (0.89, 1.18) | 0.99 (0.87, 1.13) |  | 1.24 (0.60, 2.55) | 1.06 (0.52, 2.18) |
| *P*, interaction with sex | 0.51 | 0.29 |  | 0.81 | 0.88 |
|  |  |  |  |  |  |
| Body mass index-for-age Z^4^ |  |  |  |  |  |
| Overall | 0.98 (0.89, 1.08) | 1.02 (0.93, 1.11) |  | 0.85 (0.51, 1.43) | 0.81 (0.49, 1.35) |
| Girls | 1.05 (0.91, 1.21) | 1.02 (0.89, 1.16) |  | 0.83 (0.37, 1.85) | 0.86 (0.38, 1.92) |
| Boys | 0.92 (0.82, 1.05) | 1.02 (0.90, 1.14) |  | 0.87 (0.45, 1.67) | 0.77 (0.40, 1.47) |
| *P*, interaction with sex | 0.29 | 0.74 |  | 0.82 | 0.59 |
|  |  |  |  |  |  |
| Waist circumference-for-age Z^5^ |  |  |  |  |  |
| Overall | 0.93 (0.83, 1.03) | 1.07 (0.97, 1.19) |  | 1.16 (0.69, 1.93) | 1.15 (0.69, 1.93) |
| Girls | 1.03 (0.89, 1.19) | 1.19 (1.03, 1.36) |  | 0.96 (0.45, 2.05) | 0.93 (0.43, 1.98) |
| Boys | 0.83 (0.71, 0.96) | 0.97 (0.85, 1.12) |  | 1.38 (0.70, 2.72) | 1.39 (0.71, 2.71) |
| *P*, interaction with sex | 0.04 | 0.02 |  | 0.33 | 0.32 |
|  |  |  |  |  |  |

^1^ Estimated between the 90^th^ and 10^th^ percentiles of the anthropometric index distribution from generalized estimating equations with the Poisson distribution. Seropositivity or past hospitalization was the dichotomous outcome and predictors included linear and spline terms for each anthropometric index. In all models, the robust sandwich estimate of the variance was used to account for intra-family correlations.

^2^ Estimates for hospitalization are restricted to IgG seropositive children.

^3^ Adjusted for age, sex, informant’s education level, and home ownership.

^4^ The multivariable model included as a covariate waist circumference-for-age Z adjusted for body mass index (BMI)-for-age Z through the method of residuals.

^5^ Adjusted for BMI-for-age Z through the method of residuals.

Supplementary Table S2. Unadjusted and adjusted prevalence ratios for IgG seropositivity and past hospitalization for dengue by anthropometric characteristics in children from Bucaramanga, Colombia, assuming linearity.

| Anthropometric characteristic | Seropositive  Prevalence Ratio (95% CI)^1^ | |  | Hospitalized  Prevalence Ratio (95% CI)^1,2^ | |
| --- | --- | --- | --- | --- | --- |
|  | Unadjusted | Adjusted^3^ |  | Unadjusted | Adjusted^3^ |
|  |  |  |  |  |  |
| Height-for-age Z |  |  |  |  |  |
| Overall | 1.02 (0.99, 1.06) | 1.02 (0.98, 1.05) |  | 1.04 (0.88, 1.24) | 1.00 (0.84, 1.19) |
| Girls | 1.04 (0.99, 1.09) | 1.04 (0.99, 1.09) |  | 1.01 (0.78, 1.32) | 1.00 (0.77, 1.31) |
| Boys | 1.01 (0.96, 1.06) | 1.00 (0.96, 1.05) |  | 1.07 (0.86, 1.34) | 1.02 (0.81, 1.28) |
| *P*, interaction with sex | 0.53 | 0.31 |  | 0.74 | 0.88 |
|  |  |  |  |  |  |
| Body mass index-for-age Z^4^ |  |  |  |  |  |
| Overall | 1.00 (0.97, 1.03) | 1.01 (0.98, 1.03) |  | 0.96 (0.84, 1.11) | 0.95 (0.84, 1.09) |
| Girls | 1.01 (0.97, 1.06) | 1.00 (0.96, 1.05) |  | 0.98 (0.80, 1.19) | 0.98 (0.80, 1.20) |
| Boys | 0.99 (0.95, 1.02) | 1.01 (0.98, 1.05) |  | 0.96 (0.79, 1.16) | 0.91 (0.77, 1.09) |
| *P*, interaction with sex | 0.33 | 0.72 |  | 0.88 | 0.62 |
|  |  |  |  |  |  |
| Waist circumference-for-age Z^5^ |  |  |  |  |  |
| Overall | 0.94 (0.87, 1.02) | 1.06 (0.97, 1.16) |  | 1.17 (0.79, 1.74) | 1.18 (0.80, 1.72) |
| Girls | 1.04 (0.92, 1.18) | 1.21 (1.04, 1.39) |  | 0.92 (0.49, 1.72) | 0.99 (0.52, 1.88) |
| Boys | 0.88 (0.80, 0.97) | 0.98 (0.88, 1.09) |  | 1.38 (0.85, 2.25) | 1.37 (0.86, 2.18) |
| *P*, interaction with sex | 0.04 | 0.02 |  | 0.32 | 0.32 |
|  |  |  |  |  |  |

^1^ Per unit of each anthropometric index, assuming linearity. Estimates are from generalized estimating equations with the Poisson distribution. Seropositivity or past hospitalization was the dichotomous outcome and predictors included linear and spline terms for each anthropometric index. In all models, the robust sandwich estimate of the variance was used to account for intra-family correlations.

^2^ Estimates for hospitalization are restricted to IgG seropositive children.

^3^ Adjusted for age, sex, informant’s education level, and home ownership.

^4^ The multivariable model included as a covariate waist circumference-for-age Z adjusted for body mass index (BMI)-for-age Z through the method of residuals.

^5^ Adjusted for BMI-for-age Z through the method of residuals.

Supplementary Table S3. Unadjusted and adjusted prevalence ratios for IgG seropositivity and past hospitalization for dengue by anthropometric characteristics in adults from Bucaramanga, Colombia, assuming non-linearity.

| Anthropometric characteristic | Seropositive  Prevalence Ratio (95% CI)^1^ | |  | Hospitalized  Prevalence Ratio (95% CI)^1,2^ | |
| --- | --- | --- | --- | --- | --- |
|  | Unadjusted | Adjusted^3^ |  | Unadjusted | Adjusted^3^ |
|  |  |  |  |  |  |
| Height, cm | 0.96 (0.90, 1.01) | 0.90 (0.83, 0.99) |  | 0.68 (0.21, 2.18) | 0.19 (0.04, 0.79) |
|  |  |  |  |  |  |
| Body mass index^4^, kg/m^2^ | 1.03 (0.97, 1.10) | 1.03 (0.97, 1.10) |  | 1.01 (0.28, 3.70) | 1.22 (0.34, 4.31) |
|  |  |  |  |  |  |
| Waist circumference^5^, cm | 0.93 (0.87, 0.99) | 0.89 (0.81, 0.98) |  | 1.53 (0.22, 10.93) | 1.09 (0.13, 9.00) |
|  |  |  |  |  |  |

^1^ Estimated between the 90^th^ and 10^th^ percentiles of the anthropometric characteristic distribution from generalized estimating equations with the Poisson distribution. Seropositivity or past hospitalization was the dichotomous outcome and predictors included linear and spline terms for each anthropometric index. In all models, the robust sandwich estimate of the variance was used to account for intra-family correlations.

^2^ Estimates for hospitalization are restricted to IgG seropositive adults.

^3^ Adjusted for age, sex, education level, and socioeconomic status.

^4^ The multivariable model included as a covariate waist circumference adjusted for BMI through the method of residuals.

^5^ Adjusted for BMI through the method of residuals.

Supplementary Table S4. Unadjusted and adjusted prevalence ratios for IgG seropositivity and past hospitalization for dengue by anthropometric characteristics in adults from Bucaramanga, Colombia, assuming linearity.

| Anthropometric characteristic | Seropositive  prevalence ratio (95% CI)^1^ | |  | Hospitalized  prevalence ratio (95% CI)^1,2^ | |
| --- | --- | --- | --- | --- | --- |
|  | Unadjusted | Adjusted^3^ |  | Unadjusted | Adjusted^3^ |
|  |  |  |  |  |  |
| Height, cm | 1.00 (1.00, 1.00) | 1.00 (0.99, 1.00) |  | 0.99 (0.95, 1.03) | 0.94 (0.90, 0.99) |
|  |  |  |  |  |  |
| Body mass index^4^, kg/m^2^ | 1.00 (1.00, 1.01) | 1.00 (1.00, 1.01) |  | 0.98 (0.92, 1.05) | 1.00 (0.94, 1.06) |
|  |  |  |  |  |  |
| Waist circumference^5^, cm | 1.00 (0.99, 1.00) | 0.99 (0.99, 1.00) |  | 1.01 (0.98, 1.05) | 1.00 (0.95, 1.06) |
|  |  |  |  |  |  |

^1^ Per unit of each anthropometric variable, assuming linearity. Estimates are from generalized estimating equations with the Poisson distribution. Seropositivity or past hospitalization was the dichotomous outcome and predictors included linear and spline terms for each anthropometric index. In all models, the robust sandwich estimate of the variance was used to account for intra-family correlations.

^3^ Estimates for hospitalization are restricted to IgG seropositive adults.

^2^ Adjusted for age, sex, education level, and socioeconomic status.

^4^ The multivariable model included as a covariate waist circumference adjusted for BMI through the method of residuals.

^5^ Adjusted for BMI through the method of residuals.
